# PReS-FINAL-2244: Ultrasound examination reveals typical alterations in joints of mucopolysaccharidosis (MPS) patients

**DOI:** 10.1186/1546-0096-11-S2-P234

**Published:** 2013-12-05

**Authors:** N Karabul, M Beck, A Solyom, E Mengel, C Lampe

**Affiliations:** 1 Villa Metabolica, Children's Hospital, University of Mainz, Mainz, Germany; 2 Pediatric Hospital, University of Pécs, Pécs, Hungary

## Introduction

Mucopolysaccharidoses (MPS) are a group of metabolic disorders caused by the absence or inadequate functioning of enzymes needed to break down glycosaminoglycans (GAG), which are essential structural elements of many tissues in the human body (bone, cartilage, cornea, skin, connective tissue). One of the main features of several of the mucopolysaccharidoses is bone damage and remodeling, along with synovial thickening, clinically manifested as coarse facies, thickened and widened bones on x-rays, and joint contractures of varying severity. These signs and symptoms can be the presenting features of the disease, and lead to clinical diagnosis when properly interpreted. The aversion to unnecessary exposure of patients to radiation and the question of which bones to examine by x-ray is understandable. MPS patients are additionally at a much higher risk of severe sequelae from anesthesia, making MRI examination more difficult. Therefore, the question of the best method by which to measure bone and joint involvement and progression in patients with established disease is a difficult one.

## Objectives

We present documentation of joint pathology recognizable by ultrasound in MPS patients, which might be useful to speed the diagnosis of disease, and serve as a readily available and harmless tool for monitoring of changes in the bones and joints of patients. This could be particularly useful for pediatric rheumatologists, for whom joint ultrasound is a well-established tool. We also propose a basic scoring system for quantification of such changes.

## Methods

We used ultrasound to examine MPS patients with joint disease, comparing the results to those typically found in inflammatory arthritis. The changes we have found in the MPS patients are illustrated by a representative case report.

## Results

From our observations, it appears that there are certain specific changes in the bones and joints of mucopolysaccharidosis patients apparent on ultrasound examination.

## Conclusion

Ultrasound is a useful and convenient method for documenting and following bone and joint disease in mucopolysaccharidosis patients. We have proposed a preliminary scoring system to follow bone and joint involvement in MPS, and will explore the clinical correlations further.

Finally, we wish to encourage that mucopolysaccharidosis be considered as a differential diagnostic possibility in children and adolescents with joint disease and contractures.

## Disclosure of interest

None declared.

